# Boomerang Sign in the Splenium of the Corpus Callosum After Vestibullar Schwannoma Treatment: Case Report and Review of the Literature

**DOI:** 10.3390/reports8030136

**Published:** 2025-08-04

**Authors:** Maciej Laskowski, Bartłomiej Błaszczyk, Marcin Setlak, Adam Rudnik, Ewa Warmuz-Uhma, Jan Herzyk

**Affiliations:** 1 Department of Neurosurgery, University Clinical Center, Faculty of Medical Sciences in Katowice, Medical University of Silesia, 40-752 Katowice, Poland; 2 Voxel S.A.—Radiodiagnostic Group, 40-514 Katowice, Poland; 3 Health Promotion and Obesity Management Unit, Department of Patophysiology, Faculty of Medical Sciences in Katowice, Medical University of Silesia, 40-752 Katowice, Poland

**Keywords:** boomerang sign, corpus callosum, magnetic resonance imaging

## Abstract

**Background and Clinical Significance**: The term “boomerang sign” refers to a boomerang-shaped area of cytotoxic edema in the splenium of the corpus callosum. It is seen as hyperintense lesions on T2-weighted images, FLAIR and DWI in MRI. No specific pathomechanism leading to these changes in the splenium have been yet found; however, authors have listed a variety of potential causes. **Case Presentation**: The case presents a 38-year-old male patient after left cerebellopontine angle tumor resection with an abnormal, increased signal intensity within the corpus callosum (boomerang sign) in FLAIR MRI sequence. In the case of our patient, unlike the patients described in the literature, the changes in the commissure persist. **Conclusions**: These lesions could be caused by several factors such as the development of cerebellar edema and subarachnoid bleeding or hypertonic salt usage while in the intensive care unit.

## 1. Introduction and Clinical Significance

A boomerang-shaped patch of cytotoxic edema in the splenium of the corpus callosum is known as the boomerang sign. According to the form and degree of splenium involvement, it can be categorized into two groups based on signal changes: an oval lesion with well-defined borders, or a diffuse, effaced outline with irregular borders involving the entire splenium [1]. Numerous factors, including infections, acute disseminated encephalomyelitis, hypoglycemia, hyponatremia, hypernatremia, antiepileptic medications, trauma, alcohol use, renal failure, and cerebrovascular accidents, have been listed in the literature as potential causes of transient splenial alterations [2].

It is important to note that the splenium is supplied by the vertebrobasilar system (VBS), while a significant portion of the corpus callosum derives its arterial supply from the carotid system [3].

## 2. Case Presentation

A 38-year-old patient was admitted to the Neurosurgery Department of a large academic teaching hospital in Katowice for resection of a left-sided Schwannoma tumor of the left cerebellopontine angle involving cranial nerve VIII (N. VIII). For several months prior to treatment, the patient had suffered from profound hearing loss in the left ear. The specialist consultant in otolaryngology (ENT) recommended a magnetic resonance imaging (MRI) scan of the head with a ponto-cerebellar (PC) protocol to rule out any cerebral pathology. The obtained images showed a tumor (Schwannoma) at the level of the cerebellopontine angle.

A clinical review of the patient revealed no significant concomitant diseases, and the patient denied having any allergies, or alcohol or drug addiction. In the neurological examination, the patient was conscious and able to communicate verbally and logically, without any deficits in the cranial nerves other than N. VIII, apart from the profound hearing loss in the left ear. Romberg’s test and cerebellar symptoms were negative.

The patient underwent surgery with a left retrosigmoid craniotomy, during which the tumor was partially removed. Massive brain swelling at the time of resection made further surgery impossible. The patient was transferred directly from the operating room to a computed tomography (CT) scan, where a slight subarachnoid hemorrhage (SAH) and brain edema were observed, without any parenchymal hematoma.

After the surgery, the patient was admitted to the intensive care unit (ICU) as mechanical ventilation support was required. Ultrasound of the optic nerve sheaths (ONSD) was within normal limits. Treatment for brain edema included the use of hypertonic saline, multimodal analgosedation for deep sedation, gastroprotective, mucolytic, prokinetic, nutritional, and anti-hemorrhagic treatments like tranexamic acid. The latter involved pharmacological measures to prevent or control postoperative bleeding, particularly in the context of the subarachnoid hemorrhage observed on imaging. To maintain satisfactory perfusion pressure measured invasively, using a sensor inserted into the brain ventricle, a continuous intravenous (i.v.) infusion of norepinephrine was started. Due to the inability to implement pharmacological prevention of venous thromboembolism, mechanical prophylaxis with compression of the lower limbs was instituted. A control CT scan revealed acute non-communicating hydrocephalus. Consequently, external ventricular drainage was implanted. Later, the patient was extubated and placed on passive oxygen therapy with intensive respiratory therapy.

A subsequent MRI was performed, which showed a nodular remnant at the left cerebellopontine angle, as well as typical postoperative changes. The MRI also revealed hyperintense changes in the commissure in the fluid-attenuated inversion recovery (FLAIR) sequence. After a period required for stabilization of the patient’s general clinical and neurological condition, the patient was transferred to the Department of Neurological Rehabilitation. During the course of hospitalization, the patient’s level of consciousness gradually deteriorated. A CT scan showed massive widening of the ventricular system, which led to the implantation of a ventriculoperitoneal (VP) shunt. The neurological condition improved following the implantation of the shunt, and the CT scan demonstrated regression of hydrocephalus.

**Figure 1:**
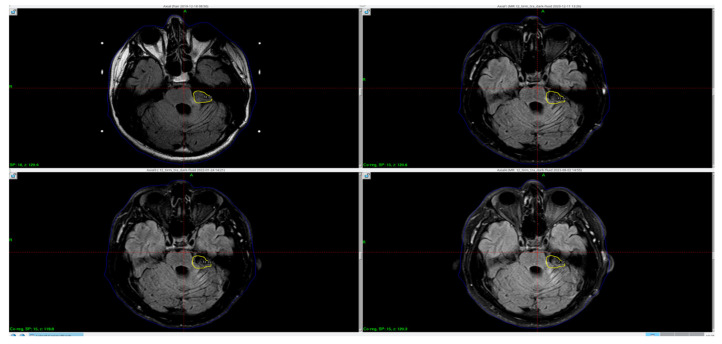
Axial MRI FLAIR images of the cerebellopontine angle following surgical resection of a tumor, demonstrating residual lesion: pre–Gamma Knife radio surgery (2019; **top left**), one-year post-treatment (**top right**), and follow-up imaging from 2022 and 2023 (**bottom**). The treatment target, corresponding to the residual mass, is delineated by the yellow isodose line as planned in the GammaPlan treatment planning system. Yellow circle—the tumor area and its remnants.

Due to the residual mass of the tumor, the patient was referred for complementary radiotherapy using the Gamma Knife method, which was subsequently performed (Figure 1).

**Figure 2:**
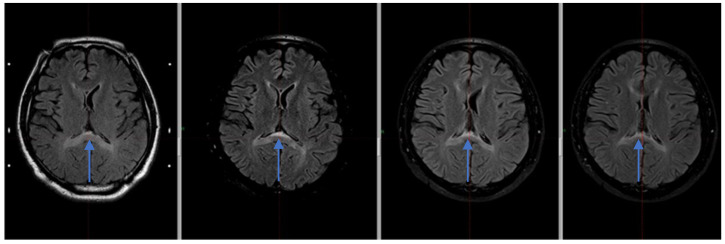
Hyperintense signal within the splenium of corpus callosum visible in the MRI FLAIR sequence in subsequent MR examinations—from the left: 12.2019, 06.2020, 12.2020, 01.2022 (blue arrows).

The MRI examination performed after Gamma Knife therapy showed also an additional linear lesion of increased signal intensity. The FLAIR sequence in the vicinity of the walls of the ventricular system, and a band of increased signal in the posterior part of the corpus callosum with moderate thinning at this height are shown in Figure 2.

## 3. Discussion

It has been reported that the “boomerang sign” may appear in patients with intracranial hypertension [4]. On diffusion-weighted imaging (DWI), patients with subarachnoid hemorrhage (SAH) may occasionally present with reversible high-signal lesions in the splenium of the corpus callosum [5]. In our case, supratentorial hydrocephalus developed due to compression of the fourth ventricle by cerebellar edema and was successfully managed with ventricular drainage. Although the patient’s condition initially improved, delayed deterioration during rehabilitation prompted further imaging, which revealed a recurrent expansion of the ventricular system.

While ischemia related to vertebrobasilar system (VBS) perfusion could be a contributing factor, this remains speculative in the absence of concurrent data on cerebral perfusion pressures. Furthermore, although no documented episode of sudden cardiac arrest occurred to suggest systemic hypoxia, the possibility of localized ischemic events cannot be entirely excluded. Blood pressure remained within target ranges during intensive care management with continuous norepinephrine infusion; however, detailed data on mean arterial pressure or cerebral autoregulatory thresholds were not recorded.

There are reports suggesting that ischemia could also lead to cytotoxic edema due to fluctuations in the arginine vasopressin system; however, hypoxia did not contribute to these changes in our patient’s case, as there was no incident of sudden cardiac arrest (SCA).

It is also worth noting that electrolyte abnormalities can result in changes in the splenium [6]. Rapid correction of acute severe hyponatremia may lead to pontine myelinolysis [7]. While our patient was in the intensive care unit, hypernatremia was used as a treatment for edema, which can also cause splenium changes visible on MRI.

Aquaporin-mediated glial swelling is another hypothesized mechanism, particularly in the context of osmotic fluctuations. The patient received hypertonic saline therapy to manage brain edema, which may have caused serum sodium fluctuations; however, no critical serum sodium derangements (e.g., severe hypernatremia or rapid correction of hyponatremia) were documented in the medical records. Nevertheless, even modest shifts in sodium levels can affect glial volume regulation via AQP4 channel activation [8]. In this context, a direct correlation between sodium trends and the timing of imaging changes could not be firmly established due to the retrospective nature of the analysis.

We acknowledge that the absence of serial intracranial pressure (ICP) measurements and detailed catecholamine dosing records limits our ability to definitively correlate clinical parameters with the imaging findings. Future studies should aim to link these variables with radiological changes to strengthen mechanistic interpretations.

Recent literature has demonstrated that lesions in the splenium of the corpus callosum, including the so-called “boomerang sign,” may not be exclusive to postoperative changes following vestibular schwannoma surgery, but can also occur in association with other posterior cranial fossa tumors such as IDH-mutant astrocytomas. In a 2025 study by De Simone et al. [9], the authors presented a detailed clinical and radiological analysis of IDH-mutant astrocytomas located in the posterior cranial fossa, emphasizing the complex interplay between tumor localization, resection strategies, and functional outcomes. Of particular interest was the observation that these tumors, despite their relatively rare location, can contribute to secondary radiological changes distant from the primary lesion site—including alterations in white matter tracts such as the corpus callosum. This supports the notion that structural and metabolic stressors related to tumor mass effect, local edema, obstructive hydrocephalus, or even postoperative hemodynamic shifts may lead to transient cytotoxic or vasogenic changes observable on MRI, including in remote regions like the splenium. The authors also emphasized the importance of maintaining an onco-functional balance, suggesting that even subtle alterations in intracranial dynamics can have measurable effects on radiological imaging and neurological outcomes. Incorporating this broader differential context strengthens the interpretation of the persistent splenial lesion in our patient, suggesting that the “boomerang sign” may reflect a nonspecific response to posterior fossa pathology and its treatment, rather than a marker of a single disease mechanism. As such, findings like ours should be evaluated not in isolation but in light of the expanding recognition of splenial involvement across various infratentorial tumor entities and treatment courses.

The boomerang sign occurs mainly in the posterior part of the corpus callosum, which may be related to its blood supply. The splenium of the corpus callosum receives its blood supply from three arteries: the anterior pericallosal artery, which is the terminal branch of the anterior cerebral artery; the posterior pericallosal artery, also known as the splenial artery; and the posterior accessory pericallosal artery. These arteries give off smaller branches that further divide into even smaller vessels, and branches from both arteries interconnect to form a network called the pericallosal pial plexus [10]. Numerous medical conditions can affect the corpus callosum because the arteries supplying the splenium are much thinner than those of the carotid system. For example, atherosclerotic plaques at the vertebrobasilar junction may cause arterial stenosis, leading to subsequent symptoms [3].

## 4. Conclusions

These differences observed in the corpus callosum on MRI could be caused by several factors. We suggest a few possible explanations that best fit our patient’s case, but we remain uncertain about the primary cause. We also do not completely rule out the possibility that these findings are coincidental.

The development of cerebellar edema and subarachnoid bleeding during surgery may be one of the causes of the MRI changes; however, other patients who experienced comparable intraoperative difficulties did not show any changes in the corpus callosum. Catecholamine use and prolonged ICU admission could be major contributors to such MRI findings, although many patients have undergone similar treatments without exhibiting changes in the commissure. We also considered the use of hypertonic saline; however, since this is a standard procedure, we have not observed such changes in other patients either. While complications during the procedure cannot be predicted, based on our experience, we suggest the prudent use of hypertonic saline as a treatment for edema.

In the case of our patient, unlike those described in the literature, the changes in the corpus callosum persist. Moreover, the patient has experienced slight double vision and memory impairment, which have gradually improved. Therefore, we will continue to perform follow-up imaging studies and investigate the underlying cause of these changes, with the aim of identifying and eliminating potential causes, as well as preventing associated neurological disorders in this clinical scenario.

We decided to describe this case because it represents a rare finding with an unclear pathomechanism and with limited literature available. If clinicians encounter similar patients, it is essential to reassure them and avoid unnecessary extensive diagnostics and treatments, such as steroids, which carry their own risks.

## Data Availability

The original contributions presented in the study are included in the article, further inquiries can be directed to the corresponding author.

## Abbreviations

The following abbreviations are used in this manuscript:

VBS	Vertebrobasilar System
N. VIII	Nerve VIII
ENT	Specialist Sonsultant at Otolaryngology
MRI	Magnetic Resonance Imaging
PC	Ponto-Cereberal
CT	Computed Tomography
SAH	Subarachnoid Hemorrhage
ICU	Intensive Care Unit
ONSD	Ultrasound of optic nerve sheaths
*i.v.*	Intra venosus
FLAIR	Fluid-Attenuated Inversion Recovery
VP	Ventriculoperitoneal
DWI	Diffusion-Weighted Imaging

## References

[1] Malhotra H.S., Garg R.K., Vidhate M.R., Sharma P.K. (2012). Boomerang Sign: Clinical Significance of Transient Lesion in Splenium of Corpus Callosum. Ann. Indian Acad. Neurol..

[2] Garcia-Monco J.C., Cortina I.E., Ferreira E., Martínez A., Ruiz L., Cabrera A., Beldarrain M.G. (2011). Reversible Splenial Lesion Syndrome (RESLES): What’s in a Name?. J. Neuroimaging.

[3] Wake-Buck A.K., Gatenby J.C., Gore J.C. (2012). Hemodynamic Characteristics of the Vertebrobasilar System Analyzed Using MRI-Based Models. PLoS ONE.

[4] Bajaj B.K., Wadhwa A., Pandey S. (2016). “Boomerang Sign”: An Ominous-Looking Finding in Reversible Maladies. Neurol. India.

[5] Toi H., Yagi K., Matsubara S., Hara K., Uno M. (2021). Clinical Features of Cytotoxic Lesions of the Corpus Callosum Associated with Aneurysmal Subarachnoid Hemorrhage. AJNR Am. J. Neuroradiol..

[6] Doherty M.J., Jayadev S., Watson N.F., Konchada R.S., Hallam D.K. (2005). Clinical Implications of Splenium Magnetic Resonance Imaging Signal Changes. Arch. Neurol..

[7] Martin R.J. (2004). Central Pontine and Extrapontine Myelinolysis: The Osmotic Demyelination Syndromes. J. Neurol. Neurosurg. Psychiatry.

[8] Giuliani C., Peri A. (2014). Effects of Hyponatremia on the Brain. J. Clin. Med..

[9] De Simone M., Choucha A., Ranalli C., Pecoraro G., Appay R., Chinot O.L., Dufour H., Iaconetta G. (2025). Astrocytomas IDH-mutant of posterior cranial fossa, clinical presentation, imaging features and onco-functional balance in surgical management. Neurosurg. Rev..

[10] Kahilogullari G., Comert A., Ozdemir M., Brohi R.A., Ozgural O., Esmer A.F., Egemen N., Karahan S.T. (2013). Arterial Vascularization Patterns of the Splenium: An Anatomical Study. Clin. Anat..

